# Impaired Motor Timing in Tourette Syndrome: Results From a Case–Control Study in Children

**DOI:** 10.3389/fneur.2020.552701

**Published:** 2020-10-29

**Authors:** Federica Graziola, Chiara Pellorca, Lorena Di Criscio, Federico Vigevano, Paolo Curatolo, Alessandro Capuano

**Affiliations:** ^1^Movement Disorders Clinic, Department of Neurosciences, Bambino Gesù Children's Hospital, Rome, Italy; ^2^Department of Neuroscience, University of Rome Tor Vergata, Rome, Italy

**Keywords:** Tourette syndrome, ADHD, motor timing, synchronization ability, finger tapping, Tower of London

## Abstract

Tourette syndrome (TS) is a neurodevelopmental disorder characterized by motor and vocal tics. Co-occurrence of attention-deficit/hyperactivity disorder (ADHD) or obsessive–compulsive disorder (OCD) is very frequent in the pediatric population as well as the presence of an impairment of the executive functions. The aim of our study was to investigate motor timing, that is, the temporal organization of motor behavior, in a pediatric population of Tourette patients. Thirty-seven Tourette patients (divided in 22 “pure” Tourette patients and 15 with ADHD) were compared with 22 healthy age- and gender-matched subjects. All subjects underwent a neuropsychiatric screening and were tested for their planning and decision-making abilities by using a standardized test, such as Tower of London (ToL). Two experimental paradigms were adopted: finger-tapping test (FTT), a free motor tapping task, and synchronization–continuation task. An accuracy index was calculated as measure of ability of synchronization. We found that “pure” TS as well as TS+ADHD showed lower scores in the FTT for the dominant and non-dominant hands than controls. Moreover, in the synchronization and continuation test, we observed an overall lack of accuracy in both TS groups in the continuation phase for 2,000 ms (supra-second interval), interestingly, with opposite direction of accuracy index. Thus, “pure” TS patients were classified as “behind the beat,” whereas, TS+ADHD as “ahead of the beat.” The performance in the finger tapping was inversely correlated to ToL total scores and execution time, whereas we did not find any correlation with the accuracy index of the synchronization and continuation test. In conclusion, here, we explored motor timing ability in a childhood cohort of Tourette patients, confirming that patients exhibit an impaired temporal control of motor behavior and these findings may be explained by the common underlying neurobiology of TS and motor timing.

## Introduction

Tourette syndrome (TS) is a childhood-onset neurodevelopmental movement disorder clinically characterized by the presence of multiple motor tics and one or more phonic/vocal tics that last for more than 1 year (1). The age at onset of TS ranges from 2 to 21 years, and the mean age is 5–7 years, with males suffering more than females in a ratio of 3–4:1 (2). Pure TS patients, referring to patients with TS without any other comorbid conditions, are relatively uncommon (3); attention-deficit/hyperactivity disorder (ADHD) or obsessive–compulsive disorder (OCD) is commonly associated (4), but other several clinical and subclinical conditions, such as explosive outbursts, conduct problems, anxiety, self-injurious behavior, and depression, could run the clinical course of the disease (5–7). Moreover, cognitive functions, and in particular, executive ones, including inhibition/attention, working memory, planning ability, and problem solving, have been reported to be impaired in TS (8), although with conflicting results (9, 10), probably because it is difficult to distinguish the role of comorbid conditions in this framework.

Time is an intriguing ability of humans and deserves several adaptive and behavioral responses to changing environment (11). Time processing is a multifaceted decoding ability of the brain depending on several factors including time intervals, reproduction of intervals, as well as estimation of a duration. Nevertheless, basic cognitive functions, such as working memory, attention, and decision making, deeply modulate and regulate time coding. Thus, although ultimate neural mechanisms are far to be completely elucidated, theories, widely accepted, on the psychological and anatomical components of interval timing are based on the neurobiological model of an “internal clock” (12), which consists of an internal pacemaker connected *via* a decision mechanism to previously important duration codes held in reference memory. Functional imaging studies in humans and lesional studies in animals pointed out the role of basal ganglia nuclei and cortico-striato-thalamo-cortical (CSTC) circuitry (13), as well as it seems that the model crucially depends on the striatal integration of oscillating cortical activity (14).

Despite its multifactorial and unknown etiology, recent studies suggest that a dysfunction of the CSTC circuits in TS leads to disinhibition and other dysfunctions in executive functioning (15, 16). Noteworthy, basal ganglia connection to the prefrontal cortex could be the neurobiological basis of impaired motor and non-motor inhibitory control, one of the key futures of TS as recently Morand-Bealieu et al. argued in a comprehensive meta-analysis (17). Distortion in motor and perceptual timing is present in many neurological and psychiatric conditions (18–21). Temporal processing has been studied also in movement disorders in both hypokinetic conditions, such as Parkinson's disease (22), as well as hyperkinetic disorders, including Huntington disease, essential tremor, and dystonia (23, 24). Findings clearly suggest that the basal ganglia network is involved in explicit motor and perceptual timing and implicit timing as well. TS has been investigated mainly for perceptual aspects of timing (23, 25, 26), meaning the ability to estimate temporal intervals measured by task of duration discrimination, duration estimation, and duration reproduction (27). Data show a reduction of accuracy on time reproduction tasks for supra-second intervals, with performance variability influenced by dopamine D2 receptor antagonists (23, 28). On the contrary, few studies have been conducted in children with TS elucidating mechanisms of motor timing. Motor timing, referring to the temporal organization of motor behavior, is a pivotal functional domain influencing the efficiency and the correctness to the context of any motor output (29). Recently, motor timing skills were investigated by Martino et al. in an adult cohort (29) and in 2011 by Avanzino et al. in a pediatric cohort (30). Nevertheless, in healthy infants, the existence of a primitive “sense” of time that changes and develops throughout childhood is well known (31), making difficult studying time in children but, likewise, even more appealing.

The ability to suppress tics, as well as to uncouple the premonitory urge sensation and tics, together with the possibility to train this capacity with appropriate techniques, reveals that motor timing plays an underestimated role in TS evaluation and management.

The aim of our study was to investigate motor timing processing in a cohort of children affected by TS with and without comorbidity compared with a healthy group of controls.

## Materials and Methods

### Subjects

Thirty-seven patients with TS diagnosed according to the Diagnostic and Statistical Manual of Mental Disorders (DSM)-5 criteria were prospectively recruited from the outpatient Movement Disorder Clinic of Bambino Gesù Children's Hospital of Rome. Inclusion criteria were (2) a defined diagnosis of TS, (3) age between 7 and 17 years old, and (4) no other neurological or general comorbidities. All TS patients were drug free from the previous 6 months before the study. Age- and sex-matched 26 healthy controls were enrolled by using a school-based recruitment call. Four of 26 healthy controls were excluded from the analysis because the neuropsychiatric screening resulted positive. The ethnicity of the entire sample was all Caucasian. Among the 37 TS patients, 15 subjects met the criteria of DSM-5 for ADHD; the remaining 22 subjects, on the other hand, had no DSM-5 diagnosis of ADHD. We consequently sub-grouped TS patients in two separate groups: TS+ADHD (*n* = 15) and “pure” TS (*n* = 22).

### Procedure

Participants were tested individually in a quiet room in the Movement Disorder Clinic of the Department of Neuroscience; tests administration was performed with clinical feasibility to avoid fatigue, with frequent breaks and chats with the children along the evaluation, in order to make them feel more comfortable and increase the focus on the activities. Each session entirely lasted from 2 to 3 h. For the computer-based tests, we used a 17-inch laptop for the presentation of stimuli and recording the responses by the participants. The LCD screen had a resolution of 1,440 × 900 pixels and a refresh rate of 60 Hz. A standard Italian keyboard was used as response keys. The background luminance of the screen was a mid-level gray measured at a viewing distance of 65 cm.

### Measures Neuropsychiatric Screening

All subjects underwent a structured neuropsychological evaluation. They were tested by either a neuropsychologist or a child neuropsychiatrist. We performed a screening for neuropsychiatric comorbidities using parent report questionnaire to assess ADHD, obsessions and compulsions disorder, anxiety disorder, mood disorder, and conduct disorder (Child Behavior Checklist [CBCL] and Conners' Parent Rating Scale [CPRS]). Children's Yale–Brown Obsessive–Compulsive Scale (CY-BOCS) was used for obsessions and compulsions severity score. Furthermore, a structural neuropsychological evaluation including (2) a non-verbal cognitive test assessing fluid intelligence (*Raven's Progressive Matrices*) and (3) a spatial problem-solving and planning task (*Tower of London* [ToL] test) was performed. All TS patients were tested for tics severity and impairment using a questionnaire interview, the Yale Global Tic Severity Scale (YGTSS). For a detailed description of the tests, see Supplementary Materials.

### Motor Timing Assessment

To assess motor timing, we used the finger-tapping test (FTT) and the sensorimotor test (SM), performed using the Inquisit 5.0 software (Millisecond^®^) downloaded on a laptop computer. Inquisit is a software widely used for experimental psychology (see www.millisecond.com for the latest research papers with Inquisit); in the Millisecond^®^ website, a test library is provided with downloadable tests (and scripts) including those used for this study. For the SM tones at 500 Hz and 70 dB, sound pressure level (SPL) (duration 50 ms) were delivered through headphones. Subjects performed a single hand task.

#### Finger-Tapping Test

We used a script by Katja Borchert of Millisecond Software^®^ (https://www.millisecond.com/download/library/v6/fingertapping/fingertapping/fingertapping.manual), adapted from the original Finger Tapping Oscillation Test part of Halstead's test battery of 1947 and later modified (32, 33). The FTT is a self-directed motor-speed test: participants have to tap with the index finger of both the dominant hand (DH) and the non-dominant hand (n-DH) as often they can within 10 s. Participants run through a mandatory number of 5 rounds (=blocks) of 10 s each. If the scores of these first 5 rounds were within 5 taps of each other, the final score was the mean of the number of taps of these 5 rounds (expressed as number of taps/10 s). On the other hand, if the scores of these 5 rounds were not within 5 taps of each other, an additional block was run, until 5 scores could be found that were within a 5-point range. The final score was the mean of these 5 scores for both the DH and the non-DH. The maximum number of rounds was 10. If no 5 scores could be found that are within a 5-point range from each other, the final score was the mean of all 10 tapping scores. After each round of testing, participants received feedback (number of taps) and got at least a 10-s rest period. After every 3 testing rounds, this resting period was increased to 60 s. We measured the final score for the DH and the final score for the non-DH.

#### Sensorimotor Synchronization Test

**Figure 1:**
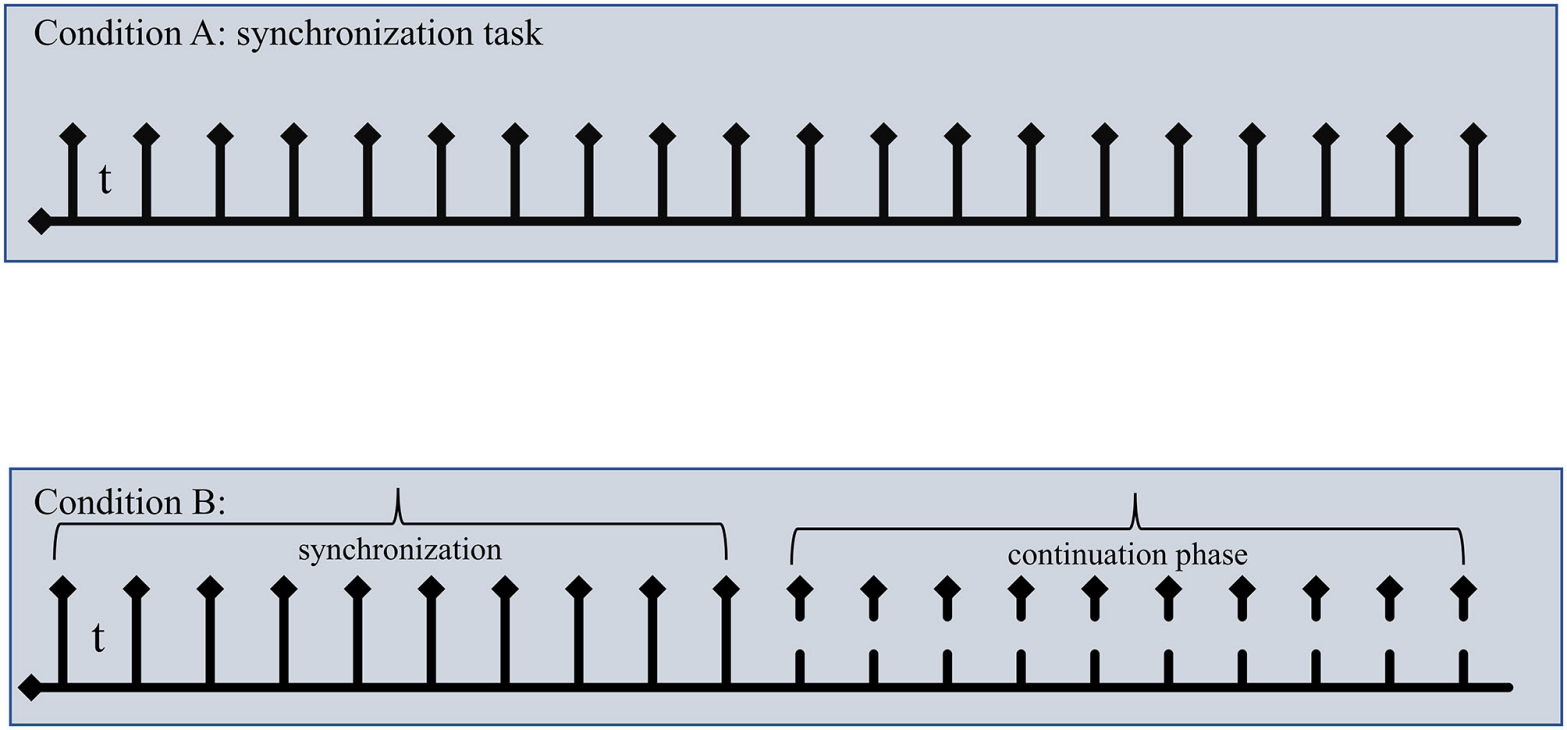
A schematic representation of sensorimotor synchronization task. Top: In Condition A, subjects were asked to tap in synchrony to “beep. Bottom: in Condition B, subjects tapped in synchrony for the first 10 taps, then beeps stopped, and subjects continued to tap at same *tempo* in the so-called continuation phase (the last 10 taps). The last 10 taps for both conditions were used in the analysis. t: intertone interval 1000 ms or 2000 ms; solid arrow indicated taps in the synchronization phase; dashed arrows indicated when beep stopped.

We used a script available from Millisecond Software^®^ Library (https://www.millisecond.com/download/library/v6/timeestimation/pacedmotortiming/pacedmotortiming.manual) implementing a Paced Motor Timing procedure described by Wittmann et al. (19). In the SM test, the participant needed to synchronize responses with a series of 20 pacer signals (beeps, 500 Hz, 50 ms durations) for two stimuli, i.e., 1000 and 2000 ms (intertone intervals). The experimental procedure consisted of two different conditions (Figure 1): (a) in Condition A (synchronization subtest), the pacer signal was played for all test trials, and the participants had to synchronize their responses (spacebar press on the keyboard) with a steady series of the pacer signals. (b) In Condition B (continuation subtest), the pacer signals were played for the first 10 tones at the beginning of the trial; after 10 tones, the beat stopped, and the participants were asked to continue tapping (spacebar keyboard press) at the same rhythm until the end without the pacer signal. The continuation tapping was performed for 20 taps. The two conditions were tested in blocked format with Condition A running first. Stimuli were sampled randomly within each condition. Variables considered in the analysis were: *tap intervals (TI)* expressed as mean (±SD) of time intervals in ms between taps as measure of tapping speed for both Condition A and Condition B. For Condition A, we also considered the *invalid responses (IRs*) as the number of motor reactions (responses after 120 ms from the onset of tone) per trial as a further measure of synchronization ability. For Condition B, we considered *TI* expressed as mean (±SD) of time intervals in ms between taps. Additionally, for both Conditions A and B, we calculated an accuracy index (Δi) as follows: *subjective time* (*TI*)/*t* where subjective time was the *TI* produced experimentally by the subject, and *t* is the *objective time*, i.e., the base interval set (1000 or 2000 ms; Δi_1000_ and Δi_2000_, respectively). This index provided the directionality of the tapping performance, being >0 if the subject was *behind the beat* and <0 if the subject is *ahead of the beat*; moreover, it was a direct measure of the magnitude of the error in reproducing the corresponding time interval. To complete the two subtests, it takes approximately 6 min. Δi was used in all statistical analysis performed.

### Statistical Analysis

Results are reported as mean ± SD. Normal distribution was assessed by Shapiro–Wilk test and Levine test for normality. Differences between means were assessed using unpaired two-tailed *t* test (where appropriate) and analysis of covariance (ANCOVA) (considering “age” as covariate) with Bonferroni *post-hoc* test for comparison between the groups, at 95% CI. For finger-tapping results, we used a two-way ANOVA, in which GROUP was analyzed as between-subjects factor, and HAND (DH vs. n-DH) as within-subjects factor. *Post-hoc* comparisons were performed using the Bonferroni *post-hoc* test. Linear least-square regression and generalized linear model (GLM) analyses were used in the selection of predictors. GLM estimation was performed by stepwise method with 95% CI. Significance was assigned for *p* < 0.05. All analyses were performed using SYSTAT software version 13.0 for Windows.

## Results

### Demographic Characteristics

Twenty-two pediatric healthy controls and 37 TS patients were included in the analysis. In the patients' group, 60% had a diagnosis of TS without any comorbid condition (pure TS, *n* = 22) and 40% with ADHD comorbidity (TS+ADHD, *n* = 15). Male to female ratio was 3:1 in both groups. All patients and healthy controls did not take any medication. Participants' ages range between 8 and 14 years old, and no statistical significance was found between the groups (ANOVA-F_[2,56]_ = 0.85; *p* = 0.43). IQ levels were 108.3 ± 13.4 in the “pure” TS group, 110.1 ± 12.1 in the TS+ADHD group, and 114.5 ± 11.7 in the control group, showing no statistical significance between the groups (ANCOVA-F_[2,55]_ = 1.77; *p* = 0.39, corrected for age). Total mean score of the YGTSS rating scale for all TS was 41.3 ± 15.4 (maximum score 100). The two TS groups did not differ for YGTSS scores (40.9 ± 14.3 vs. 41.9 ± 17.4 for “pure” TS and TS+ADHD, respectively; *t* = 0.182, df 35; *p* = 0.85, 95% CI). Total mean score of the CY-BOCS rating scale for all TS patients was 8.6 ± 1.3 (maximum score 40); particularly, the range of severity of patients with obsessions and compulsions was subclinical in 32/37 (86.5%), mild in 2/37 (5.4%), moderate in 1/37 (2.7%), and severe in 2/37 (5.4%). CBCL and CPRS scores showed statistical differences between “pure” TS and TS+ADHD in several domains including those related to ADHD comorbidity. Table 1 resumes all clinical characteristics of the population in the study.

**Table 1:** Demographic characteristics of the population in the study.

	**Controls**	**Tourette**	**Tourette plus**	***p* value**
N	22	22	15	ns
Age (mean ± SD)	11.59 ± 2.26	10.86 ± 2.25	10.8 ± 1.78	ns
Male (N)	16	16	12	ns
Female (N)	6	6	3	ns
IQ (mean ± SD)	114.5 ± 11.71	108.31 ± 13.38	110.13 ± 12.05	ns
YGTSS (mean ± SD)	–	40.90 ± 14.3	41.86 ± 17.35	ns
**CBCL-domain (%)**				
Affective problems	–	5.10	60.00	*p* < 0.001
Anxiety	–	63.64	53.33	ns
Somatic complaints	–	27.27	26.67	ns
Attention and hyperactivity problems	–	4.55	33.33	*p* < 0.001
Oppositional defiant problems	–	4.55	40.00	*p* < 0.001
Conduct problems	–	9.09	66.67	*p* < 0.001
**CPRS-domain (%)**				
Oppositional defiant problems	–	9.09	33.33	*p* < 0.001
Attention/cognitive problems	–	4.55	60.00	*p* < 0.001
Hyperactivity	–	0.00	60.00	*p* < 0.001
Anxiety	–	31.82	46.67	ns
Perfectionism symptoms	–	18.18	26.67	ns
Social problems	–	27.27	33.33	ns
Somatic complaints	–	22.73	40.00	ns
Positive ADHD index	–	0.00	100.00	*p* < 0.001

CBCL and CPRS scores are reported as percentages of patients in each domain showing a threshold score T ≥ 70. Statistical comparison between the groups is reported with corresponding p value.

*ADHD, attention-deficit/hyperactivity disorder; CBCL, Child Behavior Checklist; CPRS, Conners' Parent Rating Scale; IQ, intelligence quotient; N, number; ns, not significant; SD, standard deviation; YGTSS, Yale Global Tic Severity Scale*.

### Motor Timing

#### Finger-Tapping Test

In the FTT, DH as well as non-DH were tested. There was a statistical significance difference in mean number of taps (number of taps/trial) performed with DH between the three groups (F_[2,55]_ = 6.849; *p* = 0.002). In particular, we found a lower mean number of taps in both the Tourette groups, “pure” TS and TS+ADHD, respectively, 56.29 ± 5.78 (SD) (*p* = 0.03 *post-hoc* Bonferroni test) and 54.94 ± 7.03 (SD) (*p* < 0.01 *post-hoc* test) vs. controls, 63.28 ± 8.42 (SD). No statistical difference was found between “pure” TS and TS+ADHD. In non-DH trials, the “pure” TS group differed statistically from controls (respectively, 48.62 ± 13.31 vs. 55.81 ± 7.99; *p* = 0.043 *post-hoc* Bonferroni test), whereas no difference was found between controls and TS+ADHD (48.97 ± 8.70 vs. 55.81 ± 7.99). Considering in the model “HAND” as factor within each level (GROUP), we found that both controls and “pure” TS patients showed a statistically significant reduction with non-DH compared with DH (DH vs. non-DH, *p* < 0.01 *post-hoc* Bonferroni test). We did not find any difference in TS+ADHD between DH and non-DH.

#### Sensorimotor Test

**Figure 2:**
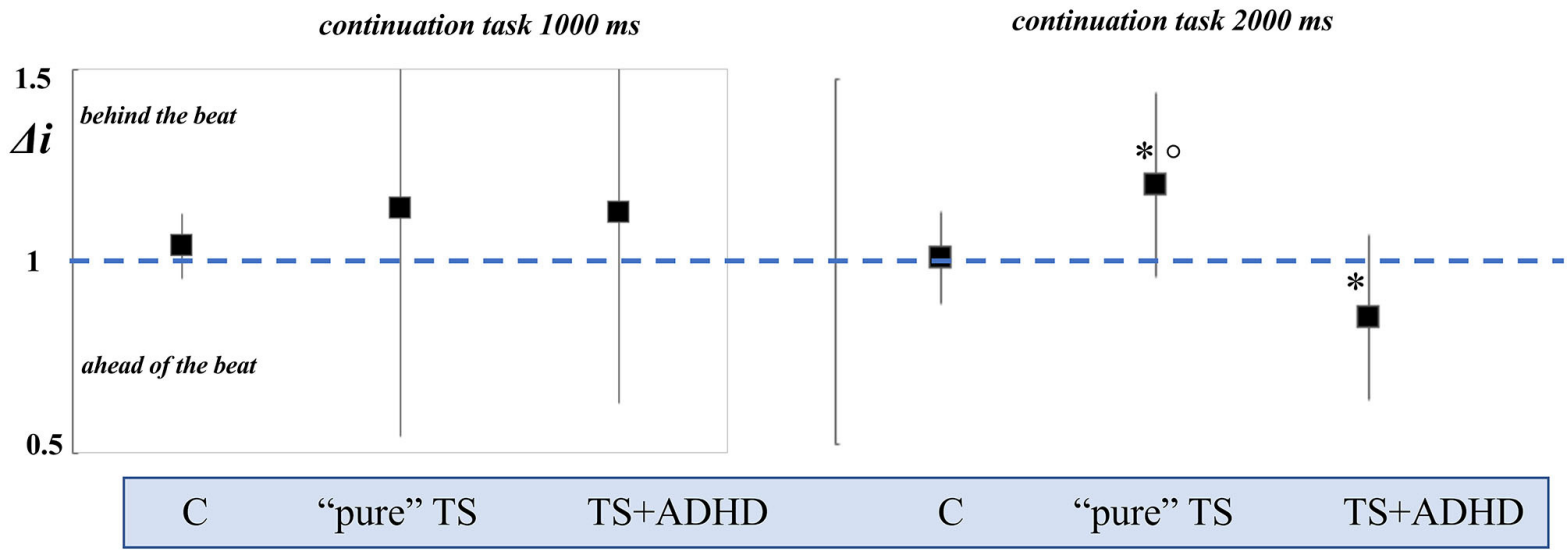
Representation of accuracy index (Δi) in Condition B (synchronization and continuation task) for *t* = 1,000 ms and for *t* = 2,000 ms. The Δi value is 1 in the iso-synchrony performance (dashed blue line); values >1 or <1 indicate the direction of continuation tapping, “behind the beat” or” ahead of the beat,” respectively. “Pure” TS and TS+ADHD patients showed a lack of accuracy in the continuation task for 2,000 ms, with opposite directions. “Pure” TS: Tourette syndrome only; TS+ADHD: Tourette syndrome plus; C: controls; ^*^p < 0.05 Bonferroni *post-hoc* test with 95% CI vs. controls; °*p* < 0.001 Bonferroni *post-hoc* test with 95% CI “pure” TS vs. TS+ADHD.

In the SM task, we tested the ability of subjects to synchronize their taps with pacer signals with or without played beep (Conditions A and B, see Materials and Methods section for details). An accuracy index (Δi) was calculated as described in the Materials and Methods section to directly assess the accuracy of synchronization along with the directionality (earlier or later) of tapping. Additionally, in Condition A, we considered IRs as adjunctive measures of synchronization. In Condition A (*paced signals*) for the interval (t) tested, 1,000 ms, all groups did not differ in a statistical manner. Δi_1,000_ values were 1.04 ± 0.04 for controls, 1.02 ± 0.05 for “pure” TS, and 1.02 ± 0.06 for TS+ADHD, indicating a good synchronization accuracy (Δi = 1). Mean number of IRs in each group per trial (reactions) confirmed the data. For the interval t, 2,000 ms, we did not find any statistical difference between the groups (F_[2,55]_ = 0.65; *p* = 0.52). Mean number of IRs confirmed the lack of difference between the groups. Table 2 shows the results expressed as TI for each group. In Condition B (*unpaced signals*), Δi_1,000_ did not differ significantly between the three groups (F_[2,55]_ = 0.20; *p* = 0.55). Conversely, in the task for 2,000 ms, both TS groups showed a statistical difference compared with controls (Δi_2,000_ = 1.01 ± 0.12; F_[2,55]_ = 14.2; *p* < 0.001), adjusted for age; in particular, “pure” TS showed a Δi_2,000_ value >1 (1.21 ± 0.25; *p* = 0.035 vs. controls; Bonferroni *post-hoc* test 95% CI), indicating a direction of synchronization “behind the beat,” whereas TS+ADHD' Δi_2,000_ was <1 (0.84 ± 0.22; *p* = 0.035 vs. controls; Bonferroni *post-hoc* test 95% CI), placing these patients significantly “ahead of the beat.” A statistical significance was found comparing “pure” TS and TS+ADHD (*p* < 0.001). Data are summarized in Table 2, Figure 2.

**Table 2:** Results of sensorimotor task.

**Sensorimotor task**				
	**Controls**	**TS only**	**TS plus**	***p*** **values**
**Condition A/synchronization** ***t*** **=** **1,000 ms**				
TI (ms) (mean ± SD)	1041.56 ± 45.66	1015.07 ± 59.34	1023.31 ± 53.73	*p* = 0.29
Invalid responses (*N*) (mean N ± SD)	2.68 ± 3.58	2.95 ± 2.40	2.93 ± 1.15	*p* = 0.90
Δi_1,000_	1.04 ± 0.04	1.02 ± 0.05	1.02 ± 0.06	*p* = 0.50
**Condition A/synchronization** ***t*** **=** **2,000 ms**				
TI (ms) (mean ± SD)	2076.16 ± 213.98	2090.56 ± 254.96	2012.56 ± 104.17	*p* = 0.50
Invalid responses (*N*) (mean N ± SD)	4.22 ± 2.04	3.86 ± 1.89	3.33 ± 0.72	*p* = 0.34
Δi_2,000_	1.04 ± 0.10	1.05 ± 0.12	1.01 ± 0.05	*p* = 0.50
**Condition B/(synchronization and) continuation** ***t*** **=** **1,000 ms**				
TI (ms) (mean ± SD)	1039.18 ± 85.02	1135.43 ± 593.51	1128.07 ± 499.61	*p* = 0.89
Δi_1,000_	1.04 ± 0.08	1.14 ± 0.59	1.13 ± 0.49	*p* = 0.89
**Condition B/(synchronization and) continuation** ***t*** **=** **2,000 ms**				
TI (ms) (mean ± SD)	2013.02 ± 252.16	2415.12 ± 506.44	1689.57 ± 447.72	*p* = 0.035^*^
				^†^*p* **<** 0.001
Δi_2,000_	1.01 ± 0.12	1.21 ± 0.25	0.84 ± 0.22	*p* = 0.035^*^
				*p* **<** 0.001^†^

Parameter for each Condition A and B and comparison between the groups are shown for each condition of the test.

TS, Tourette syndrome; TI, tap intervals; Δi, accuracy index; N, number; ms, milliseconds.

* TS only and TS plus vs. controls.

† *TS only vs. TS plus Bonferroni post-hoc test 95% CI*.

### Planning and Decision-Making Abilities

We measured planning and decision-making abilities using the standardized ToL test. Results are summarized in Table 3. Briefly, we considered the total score achieved, the mean solution time, the mean execution time, and the time of the first move (as measure of impulsivity). “Pure” TS and TS+ADHD differed from controls (*p* < 0.001) for the total score, with no effect of age in the model. As regards the total execution time, we found a statistical difference between the three groups. *Post-hoc* analysis showed a statistical significance between TS+ADHD and controls (*p* < 0.001), whereas no differences were found between “pure” TS and controls. Additionally, TS+ADHD patients showed statistically lower mean solution time values than controls (*p* = 0.045). No differences were found in the time of first move between the groups.

**Table 3:** Results of performance in the tower of London test.

	**Controls**	**“Pure” TS**	**TS+ADHD**	***p* value**
Total score	32.5 ± 2.15	26.90 ± 5.01	26.86 ± 5.51	***p*** **<** 0.001*
Solution time (s)	17.58 ± 5.79	21.58 ± 11	26.23 ± 10.8	***p*** **=** **0.045°**
Execution time (s)	6.71 ± 2.27	10.19 ± 4.28	13.94 ± 7.33	***p*** **<** **0.001**^****§****^
First move (s)	10.86 ± 4.6	12.10 ± 9.35	12.29 ± 5.4	*p* = 0.85

Data are expressed as mean ± SD. Comparison between the groups and relative p value are reported. Significant P values are displayed in bold.

ADHD, attention-deficit/hyperactivity disorder; TS, Tourette syndrome.

* Bonferroni post-hoc test with 95% CI p <0.001 pure TS and TS+ADHD vs. controls.

° Bonferroni post-hoc test with 95% CI p < 0.05 TS+ADHD vs. controls.

§ *Bonferroni post-hoc test with 95% CI p < 0.001 TS+ADHD vs. controls*.

### Correlation Analysis

Comparing the finger tapping scores with the IQ and tics severity (YGTSS), we did not find any correlation (*p* > 0.05), suggesting that tic severity did not impair *per se* motor performance. IQ and YGTSS did not correlate also with *accuracy index* for sensorimotor continuation test (Δi_2,000_). We addressed the interplay between motor timing and ToL (planning and decision-making abilities). We used a GLM to statistically test this hypothesis. In the first model, we assigned as dependent variable the “mean of finger tapping” (DH) of FTT and as independent variables “groups” (controls, “pure” TS, and TS+ADHD), the mean execution time, the total score, and interactions between them (group × mean execution time; group × total score). We found that FTT scores were statistically correlated to group as expected, whereas in the “group × mean execution time” interaction, finger tapping scores and execution time were inversely related (*p* < 0.001). In the second model, we set as dependent variable the Δi_2,000_ and as independent factors the mean execution time, the total score, and interactions between them. We did not find any significant correlation.

## Discussion

Here, we adopted two well-established experimental paradigms (32, 33), such as FTT and SM (synchronization and continuation), to study motor timing in two groups of TS patients, “pure” TS and TS+ADHD. While in free FTT, subjects tap in a freely chosen rhythm, in the synchronization and continuation task, the accuracy requires motor and perceptual timing fluctuation to replicate externally presented rhythm and memory-driven timing to continue tapping in the absence of the auditory cue (19).

In the FTT, we found that the control group scored better than both groups of TS patients when using their DH. Conversely, we found that controls scored better than “pure” TS patients also with non-DH, but not than the TS+ADHD group. In addition, controls and “pure” TS scored better with DH than with non-DH, and interestingly, this difference was not found in the TS+ADHD group. These findings deserve more clarification in light of existing data. Fine motor skills have been extensively studied in TS with conflicting results depending on simple or more complex tasks adopted (34–38) as well as on confounding factors, such as age of the experimental cohort, tic severity, and not least comorbidities. The main aim of our study was different; thus, unfortunately, we did not clearly clarify these contradictory results. On one side, we confirm the evidence that an altered organization of motor behavior occurs in TS as well as that motor skills are impaired in TS (both pure or +ADHD) in a single-hand trial; on the other hand, as expected from motor lateralization studies (30), we found a symmetric performance between DH and n-DH only in TS+ADHD and not in pure TS. The symmetry between DH and n-DH in motor performance of TS has been explained by compensatory interhemispheric plasticity mechanisms in TS (30, 39, 40). The apparent inconsistency of our results could be explained by the nature of the task (single for each hand vs. bimanual) and by other factors contributing to the development of symmetry of motor performance, such as the younger age of the cohort (34, 35). Finally, the finding that the TS+ADHD group showed a more symmetrical (poorer) performance with both hands may imply that symmetrical compensatory mechanisms can establish earlier in TS patients with comorbidity than in “pure” TS.

The main result of our study is that obtained in the synchronization and continuation task. “Pure” TS and TS+ADHD showed a poor motor timing organization with opposite performance in the 2,000 ms continuation task (supra-seconds interval), classifying “pure” TS as “behind the beat” and TS+ADHD as “ahead of the beat,” on the basis of their accuracy index. We also found that there is a lack of correlation between the timing accuracy and the tics severity scores (YGTSS), as well as with IQ scores. Martino et al. (29) recently reported motor timing performance in a cohort of adult TS patients, observing a reduced synchronization ability in the continuation condition for 2,000 ms interval. For the first time, our findings replicate this observation in a childhood cohort of “pure” TS. Furthermore, our study improves knowledge on motor timing in TS children, demonstrating that patients with comorbid ADHD scarcely synchronize as “pure” TS but with an opposite direction of accuracy (ahead of the beat) when compared with “pure” TS (see Figure 2).

ADHD comorbidity is considered a confounding factor generating conflicting results in TS studies (41). On the other hand, and strictly from a clinical point of view, ADHD accounts for at least 40% of the total comorbidity of TS patients (3, 42), and data are thought to be underestimated in several cases (43). Moreover, ADHD is one of the major components of clinical worsening of TS during lifespan (4, 44). Motor timing has been extensively studied in ADHD population with some diverging results, probably due to selection of the patients or due to different experimental conditions adopted (45–50). However, in a comprehensive review, Noreika et al. (27) concluded that both children and adults with ADHD tend to show premature responses and poor synchronization for sub-second and supra-second intervals, confirming a highly consistent pattern of motor timing abnormalities.

Motor timing is thought to be controlled by several cortical areas in conjunction with the basal ganglia and cerebellum, constituting the fronto-striato-cerebellar network (51). Additionally, functional MRI (fMRI) studies showed that sensorimotor synchronization is associated with the activation of cortical areas, such as dorsolateral frontal cortex (DLFC), inferior frontal cortex (IFC), medial frontal cortex (MFC), and supplementary motor area (SMA) [for a *review*, see (52)]. Moreover, Wiener et al. (53) found that bilateral anterior cingulate cortex (ACC), right SMA, dorsolateral prefrontal cortex (DLPFC), and inferior parietal cortex (IPC) were more involved in supra-second motor timing. After all, SMA plays a critical role in tics pathophysiology: SMA is strongly involved in tic generation in TS and also tic regulation during voluntary action (54–56). Ganos et al. (56) proposed that in TS, the right SMA seemed to act as a global inhibition mechanism in TS and was used to simultaneously stop tics and voluntary actions. Thus, SMA–striato-thalamo-cortical loop dysfunction could explain in “pure TS” patients their accuracy “behind the beat” in continuation tasks, as proposed also by Martino et al. (29). On the other hand, the opposite direction of accuracy index in TS+ADHD patients compared with pure TS leads to that other mechanisms occur. In TS+ADHD patients, it could be imagined that pre-frontal areas, such as ACC and DLPFC, may mediate the anticipatory performance for a lack of inhibitory control (impulsiveness) (49). Thus, our findings confirm once more that it is important to consider all endophenotypes in studying a puzzling complex syndrome as Tourette.

In addition, we tested patients (and controls) for their planning and decision-making abilities by performing a standardized task, such as ToL test, as measure of their “executive” function. We found that the pure TS and TS+ADHD groups compared with the control group showed lower scores in the total score, whereas the total execution time was lower in the TS+ADHD group. Planning skills refer to the capacity to organize cognitive and motor behavior in order to perform different steps needed to reach a goal (57). Termine et al. (58) showed in a small cohort of children that both pure TS and TS+ADHD patients show various impairments during the ToL test. Interestingly, we found an inverse correlation between finger tapping scores for DH and the total score and the mean execution time of the ToL test. These findings suggest, once more, that an impairment of “planning skills” in TS contributes to an impairment of the organization of motor behavior in free tapping.

However, we did not find any correlation between continuation ability and ToL performance, suggesting that not all organization of motor timing is dependent from planning ability, and this is true for both groups independently from ADHD co-occurrence.

Finally, some limitations of the study need to be addressed. First, note the small sample size of our cohort and, in particular, of the TS+ADHD group. Second, as a pure ADHD group is lacking, our results cannot be readily transferred to ADHD patients. Finally, although all our efforts have been made to minimize confounding factors, we cannot exclude intrinsic (due to referral) bias in the selection of patients.

In conclusion, we demonstrated that children with TS show an impaired motor timing organization in the free tapping and in the continuation task (tempo synchronization). Moreover, findings in TS+ADHD confirm that, at least in part, TS groups differ, signifying that ADHD co-occurrence should be considered in all studies involving TS to not exclude possible confounding effect.

## Data Availability Statement

The raw data supporting the conclusions of this article will be made available by the authors, without undue reservation.

## Ethics Statement

The studies involving human participants were reviewed and approved by Bambino Gesù Local Ethical Committee. Written informed consent to participate in this study was provided by the participants' legal guardian/next of kin.

## Author Contributions

FG, CP, and AC contributed to the conception and design of the study. CP, FG, and LD organized the database. FG and AC performed the statistical analysis. FG wrote the first draft of the manuscript. FG, AC, PC, and FV wrote sections of the manuscript and critically discussed the final version. All authors contributed to manuscript revision, read and approved the submitted version.

## Conflict of Interest

The authors declare that the research was conducted in the absence of any commercial or financial relationships that could be construed as a potential conflict of interest. The handling Editor declared a shared affiliation, though no other collaboration, with some of the authors FG, LD, and PC. The reviewer TS declared a shared affiliation, with no collaboration with some of the authors FG, LD, and PC to the handling Editor.

## Abbreviations

ADHD: attention-deficit/hyperactivity disorder
OCD: obsessive–compulsive disorder
CBCL: Child Behavior Checklist
CPRS: Conners' Parent Rating Scale
CSTC: cortico-striato-thalamo-cortical
FTT: finger-tapping test
IQ: intelligence quotient
SD: standard deviation
SM: sensorimotor test
SMA: supplementary motor area
ToL: Tower of London
TS: Tourette syndrome
YGTSS: Yale Global Tic Severity Scale.
