# Supplementary material for: Impaired Motor Timing in Tourette Syndrome: Results From a Case–Control Study in Children
Source: Front Neurol. 2020 Oct 29;11:552701. doi: 10.3389/fneur.2020.552701 (PMC7658319; doi:10.3389/fneur.2020.552701)
Supplement: Supplementary file 1 [file Data_Sheet_1.DOCX]

**Supplementary Methods**

**Parent report questionnaires**

**Child Behaviour Check List – CBCL**: a parent-report measure of child behavioural adjustment and possesses high test–retest reliability and criterion-related validity. We used the age- and sex-standardized Total Problems T score to assess child behaviour. The T score is normed for age and sex, with an average score of 50 and standard deviation of 10. Higher scores reflect poorer behavioural adjustment.

**Conners Parents Rating Scale – CPRS**: the most useful and popular rating scale for ADHD domains. Conners et al. revised this scale (Conners et al., 1998) . It contains 27 items each item is answered to on a 4-point Likert-type scale by parents (0 = never, 1 = rarely, 2 = often, and 3 = always). There are four subscales including oppositional (6 items), cognitive problem/inattention (6 items), hyperactivity (6 items) and ADHD index (12 items).

**Children's Yale-Brown Obsessive-Compulsive Scale - CY-BOCS**: a self-report tool used to aid obsessive–compulsive symptoms and diagnosis of obsessive–compulsive disorder (OCD). It is widely used throughout child, adolescent and adult psychiatry settings (Goodman et al., 2009; Bejerot et al., 2014)(Rizzo et al., 2014).

**Raven's Progressive Matrices**: a test used for measuring abstract reasoning and regarded as a non-verbal estimate of fluid intelligence. It is made of multiple-choice questions, listed in order of difficulty. This format is designed to measure the test taker's reasoning ability. The tests were originally developed by John C. Raven in 1936 (Raven and JH, 2003). In each test item, the subject is asked to identify the missing element that completes a pattern. In our sample we used the Colored Progressive Matrices or Raven Standard progressive matrices according to the age of the participant. The test is untimed but generally takes 15-45 minutes and results in a raw score which is then converted to a percentile ranking. The colored progressive matrices (CPM) is designed for children aged 5 through 11 years-of-age. This test contains sets A and B from the standard matrices, with a further set of 12 items inserted between the two, as set AB. Most items are presented on a colored background to make the test visually stimulating for participants. The standard progressive matrices (SPM) is appropriate for ages 8-65, consists of 60 problems (five sets of 12), all of which involve completing a pattern or figure with a part missing by choosing the correct missing piece from among six alternatives.

**Yale Global Tic Severity Scale (YGTSS):** is a clinical rating interview that was designed for use in studies of Tourette's syndrome and other tic disorders. The YGTSS provides an evaluation of the number, frequency, intensity, complexity, and interference of motor and phonic symptoms. (Leckman et al., 1998) (Rizzo et al., 2014).

**Spatial problem-solving and planning measures**

To asses spatial problem-solving and planning task we performed the Tower of London Test (TOL). We used a computer based three colored disks version of the Tower of London task (Krikorian et al., 1994) (Berg and Byrd, 2002). Participants are asked to arrange three coloured disks (red, green and blue) on three pegs (short, medium, high) in a specific solution pattern using the fewer the moves the possible. In the short peg one disk is allowed, in the medium peg 2 disks allowed and in the taller peg 3 disks allowed. The task consisted of 1 practice problem and 12 test problems presented sequentially and arranged in order of increasing difficulty. The aim is to reach the goal position, given the rules and number of moves. After failure to solve a problem in 3 attempts, participants are moved to the next problem in the sequence. The test required approximately 10 minutes to complete. We measured the total score (the score achieved across the whole set of test problems), the mean execution time, the mean solution time and the first move time (time between initial presentation of the target configuration and the initialization of the subject's first move, also referred as latency).
